# Do systematic reviews on pediatric topics need special methodological considerations?

**DOI:** 10.1186/s12887-017-0812-1

**Published:** 2017-03-06

**Authors:** Mufiza Farid-Kapadia, Lisa Askie, Lisa Hartling, Despina Contopoulos-Ioannidis, Zulfiqar A. Bhutta, Roger Soll, David Moher, Martin Offringa

**Affiliations:** 1 Child Health Evaluative Sciences, Research Institute, The Hospital for Sick Children, University of Toronto, 555 University Avenue, Toronto, ON M5G 1X8 Canada; 2 Systematic Reviews & Health Technology Assessment, NHMRC Clinical Trials Centre, The University of Sydney, Sydney, Australia; 3 Alberta Research Centre for Health Evidence, Department of Pediatrics, University of Alberta, Edmonton, Canada; 4 Department of Pediatrics, Division of Infectious Diseases Stanford University School of Medicine, Stanford, USA and Meta-Research Innovation Center at Stanford (METRICS), Stanford, USA; 5 University of Vermont College of Medicine and Cochrane Neonatal Review Group, Burlington, USA; 6 Centres for Practice-Changing Research, Ottawa Hospital Research Institute, Ottawa, Canada

**Keywords:** Systematic reviews, Pediatrics, Methods, Reporting guideline

## Abstract

**Background:**

Systematic reviews are key tools to enable decision making by healthcare providers and policymakers. Despite the availability of the evidence based *Preferred Reporting Items for Systematic reviews and Meta-Analysis* (PRISMA-2009 and PRISMA-P 2015) statements that were developed to improve the transparency and quality of reporting of systematic reviews, uncertainty on how to deal with pediatric-specific methodological challenges of systematic reviews impairs decision-making in child health. In this paper, we identify methodological challenges specific to the design, conduct and reporting of pediatric systematic reviews, and propose a process to address these challenges.

**Discussion:**

One fundamental decision at the outset of a systematic review is whether to focus on a pediatric population only, or to include both adult and pediatric populations. Both from the policy and patient care point of view, the appropriateness of interventions and comparators administered to pre-defined pediatric age subgroup is critical. Decisions need to be based on the biological plausibility of differences in treatment effects across the developmental trajectory in children. Synthesis of evidence from different trials is often impaired by the use of outcomes and measurement instruments that differ between trials and are neither relevant nor validated in the pediatric population. Other issues specific to pediatric systematic reviews include lack of pediatric-sensitive search strategies and inconsistent choices of pediatric age subgroups in meta-analyses. In addition to these methodological issues generic to all pediatric systematic reviews, special considerations are required for reviews of health care interventions’ safety and efficacy in neonatology, global health, comparative effectiveness interventions and individual participant data meta-analyses. To date, there is no standard approach available to overcome this problem. We propose to develop a consensus-based checklist of essential items which researchers should consider when they are planning (PRISMA-PC-Protocol for Children) or reporting (PRISMA-C-reporting for Children) a pediatric systematic review.

**Summary:**

Available guidelines including PRISMA do not cover the complexity associated with the conduct and reporting of systematic reviews in the pediatric population; they require additional and modified standards for reporting items. Such guidance will facilitate the translation of knowledge from the literature to bedside care and policy, thereby enhancing delivery of care and improving child health outcomes.

## Background

Historically, children have been underrepresented, and often excluded, from clinical research [1]. The studies that have been done in children have shown limited quality across the hierarchy of research evidence as compared to studies in adults [2]. This leaves pediatric populations, and end users such as health care providers and policy makers with a knowledge gap that has led to treatments based on extrapolations of safety and effectiveness from adult data, neglecting the complexities surrounding intervention exposure in children [1, 3, 4], and leading to under- or over-treatment with potential lifelong consequences. This scarcity of research also affects evidence synthesis. A survey of the quality and quantity of evidence on child relevant Cochrane systematic reviews showed limited number of relevant systematic reviews in children and even fewer provide representative information by age-group strata [5].

In modern health care, systematic reviews and meta-analyses are key tools in decision making by healthcare providers and policy makers, as they guide patient management and provide insight towards further research required. However, the methodological quality of systematic reviews has been questioned, even of those published in high impact factor journal [6, 7]. Concerns have been raised regarding weaknesses in their design, conduct, and reporting, which impair their applicability to practice and policy guidelines [6, 7]. In 2009, the PRISMA statement was published to provide guidance to authors, peer reviewers, and journal editors on elements for optimal reporting of systematic reviews and meta-analyses of treatment comparisons in order to maximize the transparency, replicability, and quality of such studies [8]. In the 2 years following the publication of PRISMA statement, a modest improvement in completeness of reporting of systematic reviews and meta-analyses in sub-specialty journals was reported [9], not surprisingly primarily in studies published in journals endorsing the PRISMA statement [10].

For any research project, the reporting stage is too late to correct errors in design and conduct. The protocol for a systematic review serves as the foundation of the study’s conduct and reporting. However, systematic review protocols are seldom published and only 1 in 10 non-Cochrane systematic review mentions working from a protocol to complete the review [11]. Systematic review protocols permit readers to determine whether unplanned decisions, not otherwise documented in a published protocol, were introduced including change of primary outcomes or reporting of statistically significant outcomes [12]. Unplanned reporting of systematic review results may lead to biased conclusions and inappropriate decisions by end-users. In contrast, a published or registered protocol allows a comparison of reported review methods with the planned methods. Pre-publication of protocols also avoids unnecessary duplication of efforts and provides transparency for possible publication bias in the field [13]. To address these problems, an international Prospective Register of Ongoing Systematic Reviews (PROSPERO) was launched in February 2011, and the PRISMA-P (Preferred Reporting Items for Systematic Reviews and Meta-Analyses - Protocol) statement providing an evidence-based guidance on 17 key items to be reported in systematic reviews and meta-analysis protocols was published in 2015 [14].

Despite these positive developments, a large number of current pediatric systematic reviews lack clarity in reporting and therefore offer limited or biased evidence for recommendations, partly due to the existence of guidelines that are not specific to child health research.

Evaluations of systematic reviews in some pediatric subspecialty fields, e.g., oncology [15] and urology [16], reported low quality. Furthermore, compared to adult research, a scarcity of high quality evidence in pediatric-specific research exists for both randomised controlled trials (RCTs) and systematic reviews [2]. Out of the 4520 systematic reviews that have been registered in PROSPERO in February 2015, less than 10% were on the pediatric population aged 0–18 years. An evaluation of “child relevant” systematic reviews found that only about half of the reviews intended to included data from children [17]. Unfortunately, where data have been synthesized, results are most often uninformative and unreliable, due to a high variability in systematic reviews and meta-analytic methods applied in child health research. Cautious interpretation of inconclusive findings from such pediatric systematic reviews should be made in order to assist physicians best communicate the available evidence with their patients and make the best informed decision during routine clinical practice. While several of the limitations identified in pediatric systematic reviews might also reflect limitations of evidence in the original pediatric RCTs, nevertheless, there are also several areas of improvement for the pediatric systematic reviews per se, which we analytically discuss below.

In this paper, we discuss methodological issues specific to the design, conduct, analysis and reporting of pediatric systematic reviews.

## Discussion

### The ‘PICOSS’ for pediatric specific systematic reviews

A systematic review typically starts with identifying the PICO (**P**opulation, **I**ntervention, **C**omparator and **O**utcomes) for the research question. Two additional elements need particular attention in pediatric systematic reviews: the **S**earch Strategy, and any planned **S**ub-group analysis. Without a specific and precise research question, a review lacks direction and is prone to subjective interpretation. Although there is limited empirical appraisal of the quality of systematic reviews for most of the pediatric subspecialties, as few as 27% of the systematic reviews in pediatric urology had a research question explicitly and properly stated [16]. A well-defined research question is also critical for developing the search strategy and planning the statistical analysis, in particular any subgroup analyses. We describe below the consequences unique to each of these issues in pediatric systematic reviews.

#### Population

A fundamental decision at the outset of a systematic review is whether to focus on pediatric population only, or to include both adult and pediatric population. This decision is often based on biological plausibility as children go through different developmental stages. A survey of the Cochrane database found that in 35% of the pediatric relevant systematic reviews, the age criteria of the intended population in a systematic review was not specified [17]. Furthermore, 80% of the intended pediatric systematic reviews actually included pediatric sub-populations only, while 63% of the intended “mixed populations” reviews actually included both adult and pediatric trials and/or mixed trials. However, this might also reflect the paucity of pediatric RCTs to be included in a systematic review rather than limitations of the systematic reviews. Substantial differences exist between systematic reviews with respect to how infants, children, adolescents, and adults are defined. This difference may be due to the variation in age categories used in primary studies and insufficient attention to the importance of age groupings for public health relevance [18]. While PRISMA recommends to explicitly state the patient population being addressed and their defining characteristics such as disease and the setting of care considered, justifying the eligible pediatric age range and age group(s) selected for the systematic review, addressing potential age related differences in intervention effects are critical reporting elements in pediatric systematic reviews.

#### Intervention

It is crucial to consider the growth and developmental trajectory in describing interventions in pediatric systematic review. A critical requirement of intervention description for pediatric systematic review is the justification of the suitability of the interventions to the targeted pediatric age group(s), addressing age related differences in dose, duration, strength, route of administration and bioavailability. For example, dosing for vitamin A supplementation in neonates would be different from infants 1 to 6 months of age, due to the variation in pharmacokinetics and pharmacodynamics in different age strata as well as other contextual factors such as supplementation of mothers during lactation, birth weight and timing of vitamin A supplementation, either within the first 48 to 72 h or later [19]. However, limited data from primary pediatric studies preclude subgroup analyses for the different doses of interventions for targeted pediatric age sub-groups, resulting in “false negative results” (failure to detect true beneficial effects due to very small subgroups). Such limitations should be acknowledged in a systematic review. While PRISMA recommends to explicitly state the interventions or exposure of interest, justifying an intervention for a pediatric age range and for specific age subgroups, and providing a rationale for extrapolation or manipulation of adult intervention are critical reporting elements in pediatric systematic reviews.

#### Comparators

Determination of an appropriate comparator in pediatric systematic reviews is critical, both from the policy and patient care point of view. Placebo is a common comparator in pediatric systematic reviews as it is in pediatric clinical trials [20]. Comparators need to be justified since the “standard of care” may involve an off-label or unlicensed drug, which often does not have strong evidence for safety or effectiveness in the pediatric population [21]. This issue is further complicated in pediatric systematic review by the fact that placebo response rates in drug trials appear to be higher in children and adolescents in comparison with adults [22, 23], and consequently, pooled response rates are higher in children and adolescents than those known for adults with similar conditions [24]. This higher placebo response in children also suggests that extrapolating drug efficacy in evidence synthesised in adults to children may result in underestimating the placebo response and overestimating drug efficacy [24]. Furthermore, all alternate interventions given in routine clinical practice as a standard of care should be considered when planning a pediatric systematic review. While PRISMA recommends to clearly report the comparator (control) group, such as standard of care, drug, or placebo, an explanation for the choice of comparison group and, if applicable, evidence for the active comparator used (for systematic reviews of RCTs) or “standard of care” for the pediatric age sub-groups are important reporting elements in pediatric systematic reviews.

#### Outcomes

The selection of outcomes a priori should be based on sound knowledge of the disease trajectory and children’s growth and development. Relevant short-term and long-term outcomes may differ for children and adults, and similar outcomes may be assessed using different measurement tools. While efforts have been made to develop Core Outcome Sets (COS) for several health conditions, many of these COS are not age specific. As health outcomes in children are different from adults, the methodology behind selecting and measuring valid, responsive and feasible outcomes for pediatric research are key. Core Outcome Sets for pediatric health conditions are a new opportunity to attain high research standards [25, 26]. Recognition that the benefits and harms of treatment for children may unfold across subsequent decades of life may influence the choice of outcomes and study designs to be included in a systematic review. A recent systematic review suggests paucity and limitations of the evidence investigating the long-term outcomes of recommended interventions in children with attention deficit hyperactivity disorder [27]. Furthermore, use of short and long term outcomes in pediatric clinical research was often heterogeneous and ad hoc, limiting synthesis of evidence in the pediatric population [28, 29]. This heterogeneous selection of outcomes presents a challenge when assessing the totality of evidence based on different trials (in the context of meta-analysis). The synthesis of the accumulated evidence from different trials into systematic reviews is impaired by the use of outcomes and outcome measurement instruments that are neither qualified nor validated in pediatric sub-populations [29], with no standard approach available to overcome this problem in a systematic review. Outcomes of interest for a systematic review, but for which no data could be identified in the primary studies should be transparently reported in the systematic review as “remaining knowledge gaps.” A descriptive analysis of child relevant systematic reviews in the Cochrane database found that a primary outcome was identified in 72% of pediatric systematic reviews [5]. However, this number varied substantially between sub-specialities, from 27% in injuries to 90% in infectious diseases [5]. A priori identification of outcomes is needed to avoid outcome reporting bias in systematic reviews [12], acknowledging that a priori primary outcome for the systematic review, might not have been a primary outcome for the original studies. While PRISMA recommends to clearly specify the outcomes of the intervention being assessed, explaining the clinical relevance of the selected outcomes (benefits and harms) for the pre-specified pediatric age group(s) and the validity, feasibility and responsiveness of the outcome measures for the pre-specified pediatric age group(s) are important reporting elements in pediatric systematic reviews.

#### Search strategy

Given the vast amount of research (e.g., currently 24 million citations of biomedical literature in PubMed alone), clinicians and researchers seeking research studies for targeted pediatric age subgroups, for example adolescence or neonates, need to target their literature searches so that the retrieved information is relevant to their patient population [30]. Moreover, bibliographic databases have varying definitions of “child” and other age-based terms, inconsistent with other databases. Titles and abstracts of pediatric specific research describe the age of the study population very inconsistently, if at all. Pediatric systematic reviews have been reported to be particularly weak in terms of the comprehensiveness in their search to identify primary studies [31]. Consequently, search filters have been developed, tested and validated to capture pediatric studies [30, 32, 33]. A study by Kastner et. al. determined the retrieval characteristics of age-specific search terms in MEDLINE for pediatric and neonatal medicine and found that highest sensitivity and specificity was achieved by a combination of MESH terms and key words (pediatric medicine - 98 and 81.2% and neonatal medicine - 95.3 and 83.6%, respectively) [30]. While PRISMA recommends to present the full electronic search strategy, description of any tested pediatric search filters used in the systematic review including sensitivity and precision of the search filters for retrieving child health systematic reviews would improve the appropriateness of search methods used in the pediatric systematic review.

#### Statistical (Subgroup) analysis plan

Within the wide age range of 0–18 years, one can expect differing response to interventions and a wide range of delivery methods. Authors should analyse age groups where there may be important similarities or differences in terms of physiological and psychosocial development as well as delivery strategies. However, only 13% of the authors of pediatric relevant systematic reviews planned to conduct a subgroup analysis based on age, of which 60% (31/52) conducted the planned subgroup analysis [17]. Moreover the use of overlapping pediatric age subgroups has been reported [18]. In the “pediatric only reviews”, only 23% provided age sub-group analysis [18], and age categories used in these analyses varied substantially even for similar interventions and health conditions [17, 18]. While synthesising data increases statistical power and allows for more precise effect estimates of treatment comparison, a meta-analysis covering the whole of the pediatric population may be unsuitable when studies are too heterogeneous in terms of the pediatric age categories they include. These important issues will only be able to be fully assessed when systematic reviews in children use consistent, clearly reported age sub-groups, which is heavily dependent on the use of consistent age sub-groups in primary research. While PRISMA recommends to report any subgroup analyses undertaken, in pediatric systematic reviews, it is also critical to include description of handling data from primary studies that include both adult and pediatric population but without separate subgroup analysis for pediatric population were dealt in the analysis (where applicable).

### Special issues in pediatric systematic reviews

#### Systematic reviews in global child health

The Millennium Development Goals (MDGs), established in 2000, committed the global community to achieve a set of targets by 2015, relative to baseline figures of 1990. At the core of the MDGs are MDG 4, which calls for a reduction of under-5 child mortality by two-thirds, and MDG 5, which calls for a reduction of maternal mortality by three-quarters and universal access to reproductive health [34]. Although some progress has been achieved, global progress is off-track and rates of reduction in maternal and child mortality in many developing countries are much slower than anticipated [35, 36]. Therefore, understanding the causes of child mortality and the effectiveness of the preventive and treatment interventions has become one of the main interests of the global health community. Much of the development and implementation of programs/interventions addressing Maternal and Child Health (MNCH) and preventable deaths over the last decade has been achieved by using existing knowledge and evidence [37–42], guided by systematic reviews and meta-analyses to determine the most effective interventions and delivery strategies. This evidence has also been compiled into consensus documents [43]. However, it is evident that the current status of global guidance based on systematic reviews of the evidence also highlights major shortcomings in relation to quality of the evidence; i) due to limited resources, many of the studies are of limited sample size and insufficiently powered for key outcomes; ii) included studies do not always provide disaggregated information of clinical relevance; iii) risk of bias pertaining to source of funding for included trials was not evaluated in the child health systematic reviews [44]; iv) diversity of study settings and contexts that produces enormous confounding and limits generalizability of findings, requiring routinely explored in RCTs from different country-settings (more developed vs. less developed countries [45]; v) mortality is a rare outcome of an intervention, and the introduction of proxy indicators of mortality creates uncertainty over the true effects on mortality and causes ambiguous conclusions [46]. Despite the rigorous nature of the global guidance process led by the World Health Organization through its guideline development process [47], in many instances the quality of the evidence submitted and collated, leaves much to be desired.

#### Individual participant data systematic reviews in paediatrics

Traditionally systematic reviews have included meta-analysed data that have been sourced from published studies where such data have already been ‘aggregated’ by the researchers and presented in tables, figures and text. Whilst this is certainly an improvement on merely selecting published literature in a non-systematic way and synthesising the results of a collection of studies through descriptive methods, ‘aggregate data’ meta-analyses have several important limitations. These include often not being able to access all data collected in all trials, data being available only in the published (age) sub-group categories and the use of different child health outcome definitions across the included trials.

The use of line-by-line raw data from individual trial participants, sourced directly from the trialists, can overcome many of these problems. This is known as individual participant data, or IPD, meta-analysis. This type of meta-analysis is used commonly in cancer and cardio-vascular settings but has only recently been used to address pediatric questions [48]. IPD meta-analysis is particularly useful in the commonly seen problem in pediatrics whereby each trial has used different age cut-points for presenting sub-group analysis. For example, in trials of neonatal therapies some will present results sub-grouped by gestational age </≥ 26 weeks, others </≥ 28 weeks and others still </≥ 32 weeks. With access to the gestational age of every infant in every study that is possible with IPD, consistent age sub-group analyses can be conducted using the maximal amount of available data [48]. Similarly, the ability to group participants accurately according to intervention-level characteristics (e.g. dosage/exposure, level of background care) enables significantly greater data utilisation. Another limitation of systematic reviews using aggregate data is that different trials often use different definitions of the same outcome (e.g. neonatal chronic lung disease) making data synthesis problematic. Again, with access to the various components of an outcome (e.g. type and duration of respiratory support, additional oxygen challenge tests etc.), common definitions can be applied across all trials to assess whether or not such definitional differences impact on the robustness of the meta-analysis conclusions. For all these reasons, IPD meta-analysis should be considered more often when addressing paediatrics questions.

#### Comparative effectiveness systematic reviews in pediatrics

Pediatric systematic reviews on the comparative effectiveness of therapeutic or preventive interventions need to consider clinically relevant outcomes for each age subgroup that may be eligible for the intervention that cover both efficacy and harms. Harms outcomes may include severe adverse events, discontinuations due to adverse events and mortality, in addition to organ-system specific adverse events (e.g., renal adverse events) or individual specific adverse events (e.g. vomiting/diarrhea).

Given that pediatric randomized evidence is often sparse and inconclusive on its own, evidence on the comparative effectiveness/safety of interventions based on pediatric RCTs should be systematically evaluated in the context of the broader existing evidence, e.g. evidence generated in adults [3, 4]. It may be also worthwhile to consider existing evidence from comparative effectiveness studies using routinely collected data (e.g. studies based on Electronic Health Record data or registries, etc. [49, 50]). This may make pediatric systematic reviews more clinically useful for policy makers and guideline developers who are asked to appraise the totality of the available evidence (randomized and non-randomized) to make policies and guideline recommendations. However, due caution is needed in extrapolating from adults to children and in appraising the validity of observational data for effectiveness purposes.

The agenda of pediatric systematic reviews should also be broad enough to target all possible interventions (e.g. medical, surgical and/or non-medical/surgical Interventions) and/or all possible outcomes that could be considered during routine clinical practice and management of pediatric patients [51]. Whenever possible, network meta-analyses should be incorporated in pediatric systematic reviews to quantitatively appraise the wider agenda of all available therapeutic options [52].

Finally, the interpretation of the comparative effectiveness/safety of pediatric interventions should be based on a priori decision on what would be considered a clinically significant absolute or relative difference in a pediatric population. Given the dearth of pediatric evidence, it is common to have mostly or entirely non-statistically significant summary effects in pediatric systematic reviews and meta-analyses. This should not be considered as pinpointing simply that the compared interventions do not differ. Interpretation of results showing non-statistically significant differences between compared interventions should be cautiously done and discussed in the context of the remaining uncertainty based on the 95% confidence intervals around the estimated effect-sizes (e.g., when no statistically significant difference in treatment effect was found, based on the limited amount of evidence it could not be excluded that the experimental intervention could have been up to X% worse or up to Y% better than the compared intervention).

#### Systematic reviews in neonates

Neonatal-Perinatal Medicine has a long history of production of systematic reviews beginning with the landmark book, “Effective Care of the Newborn Infant”, edited by Sinclair and Bracken [53], to the current Cochrane Neonatal Review Group, which has published over 300 systematic reviews in Neonatal-Perinatal Medicine. Neonates represent a unique population. Outcomes, particularly for preterm infants, are distinct from virtually every outcome measure in other systematic reviews. This includes complications of prematurity, such as bronchopulmonary dysplasia, intraventricular hemorrhage, necrotizing enterocolitis and retinopathy of prematurity. The neonatal population in particular is prone to significant developmental problems after being treated for critical illness in the newborn period. A classic example of this would be the use of steroids postnatally to prevent or treat chronic lung disease [54–56]. Short-term outcomes were extremely beneficial (reduction in oxygen) but long-term outcomes were particularly concerning (increase in cerebral palsy). Without careful analysis of the unique attributes of the at-risk population, as well as outcome measures that reflect both the short-term and long-term complications, such reviews lack meaning to the neonatal community. This has been managed by having specialty groups, such as the Cochrane Neonatal Review Group, but clearly standards need to be set worldwide for authors who attempt to do these types of analyses in this vulnerable population.

### Systematic reviews on pediatric topics need special methodological considerations

Given the methodological differences identified in pediatric systematic reviews, it is evident that they require additional and modified standards with regard to design, conduct and reporting. Differences have been identified in terms of consideration of the pediatric population and targeted pediatric age group(s), intervention, comparator, relevant, valid and feasible outcomes in children. Here, decisions need to be based on the biological plausibility of differences in treatment effects across the developmental trajectory in children. Further issues specific to pediatric systematic reviews include lack of pediatric-sensitive search strategies and inconsistent choices of pediatric age subgroups in meta-analyses.

The need for pediatric-specific items in reporting guidelines was also evident in an international consensus meeting on Standard Protocol Items for Randomized Trials in Children (SPIRIT-C) and Consolidated Standards of Reporting Trials in Children (CONSORT-C) in Toronto in 2014 that agreed on 8 and 14 pediatric-specific extension items, respectively, for the design and conduct (SPIRIT-C) [57] and reporting (CONSORT-C) [58] of pediatric clinical trials. In the same meeting of key stakeholders and experts of pediatric clinical trials, an urgent need of guideline to conduct pediatric-specific systematic reviews was highlighted. Therefore, as the need to enhance high quality primary research in children is more pronounced than ever, there is for a scientific and ethical obligation for standardisation of research protocols, research practice, and reporting standards for pediatric systematic reviews. In addition to the methodological considerations generic to all pediatric systematic reviews, special considerations are required for reviews in neonatology, childhood cancer, global health, community-based interventions and individual participant data meta-analyses.

To date, the available reporting guidelines, including PRISMA (and PRISMA-P), do not adequately cover the complexity associated with systematic reviews in the pediatric population. These child health unique aspects of pediatric systematic reviews, some of which have been addressed in various recent PRISMA extensions (e.g. IPD [59], Network Meta-Analysis [60] and Equity [61]) and in the SPIRIT-C 2014 Statement [57], are summarized in the Table 1. It has become clear that they play a decisive role throughout the design, conduct and reporting of high quality pediatric systematic reviews.

**Table 1:** Potential modification and extension items beyond the current PRISMA-P and PRISMA for child-centric systematic reviews

Section/topic	#	PRISMA-Original Item Potential modifications* for PRISMA-Children are bolded	PRISMA-Children Potential extension items
Title			
Title	1	Identify the report as a systematic review, meta-analysis, or both **for pediatric population as a focus of review with age group stated, if applicable.**	
Abstract			
Structured summary	2	Provide a structured summary including, as applicable: background; objectives; data sources; study eligibility criteria, **including specifying targeted pediatric age groups,** interventions; **primary and secondary outcomes,** study appraisal and synthesis methods; results; limitations; conclusions and implications of key findings; systematic review registration number.	2a. If a systematic review includes both adults and children, describe a subgroup analysis for the pediatric population(s) in the methods and results of the abstract 2b. Abstract report broad search strategy related to pediatric population 2c. Abstract describes applicability or limits of applicability of results to the pediatric group and sub-group(s)
Introduction			
Rationale	3	Describe the rationale for the review in the context of what is already known.	3a. Describe potential for extrapolation of evidence from adult data, or why extrapolation is not considered possible 3b. Describe any hypotheses that relate to particular pediatric population or pediatric subgroups.
Objectives	4	Provide an explicit statement of questions being addressed with reference to **targeted pediatric age group(s),** interventions, comparisons, outcomes, and study design (PICOS).	
Methods			
Protocol and registration	5	Indicate if a review protocol exists, if and where it can be accessed (e.g., Web address), and, if available, provide registration information including registration number.	
Eligibility criteria	6	Specify study characteristics (e.g., PICOS, length of follow-up) and report characteristics (e.g., years considered, language, publication status) used as criteria for eligibility, giving rationale.	6a. Participants: Justify the eligible pediatric age range and age group(s) selected for the systematic review, addressing potential age related differences in intervention effects 6b1. Intervention: Justify an intervention for pediatric age range and age groups 6b2. Provide rationale for extrapolation or manipulation of adult intervention, if any 6c. Comparison: Explanation for a choice of comparison group and, if applicable, evidence for active comparator (for systematic reviews of RCTs) or standard of care for pediatric population and/or sub-groups 6d1. Outcomes: State all short and long term outcomes addressed for pediatric population and define them in detail. State whether they were primary/main or secondary/additional outcomes 6d2. Outcomes: Explain the clinical relevance of the selected outcomes (benefits and harms) for the pre-specified pediatric age group(s) 6d3. Outcomes: Explain the validity, feasibility and responsiveness of the outcome measures for the pre-specified pediatric age group(s)
Information sources	7	Describe all information sources (e.g., databases with dates of coverage, contact with study authors to identify additional studies) in the search and date last searched.	
Search	8	Present full electronic search strategy for at least one database, including any limits used, such that it could be repeated.	8a. Describe the broad search strategy and terms (including database specific MeSH terms for pediatric population) used to address the pediatric population 8b. Describe any tested pediatric search filters used in the systematic review including sensitivity and precision of the search filters for retrieving child health systematic reviews
Study selection	9	State the process for selecting studies (i.e., screening, eligibility, included in systematic review, and, if applicable, included in the meta-analysis).	
Data collection process	10	Describe method of data extraction from reports (e.g., piloted forms, independently, in duplicate) and any processes for obtaining and confirming data from investigators.	
Data items	11	List and define all variables for which data were sought (e.g., PICOS, funding sources) and any assumptions and simplifications made.	
Risk of bias in individual studies	12	Describe methods used for assessing risk of bias of individual studies (including specification of whether this was done at the study or outcome level), and how this information is to be used in any data synthesis.	12a. For each included trial in a systematic review, indicate (a plan to include) the source of financial support (such as Government, Academia or Industry), if any, for the trial(s).
Summary measures	13	State the principal summary measures (e.g., risk ratio, difference in means).	
Synthesis of results	14	Describe the methods of handling data and combining results of studies, if done, including measures of consistency (e.g., I^2^) for each meta-analysis.	14a. Describe how studies (that include both adult and pediatric population but) without separate subgroup analysis for pediatric population were dealt in the analysis (where applicable)
Risk of bias across studies	15	Specify any assessment of risk of bias that may affect the cumulative evidence (e.g., publication bias, selective reporting within studies).	
Additional analyses	16	Describe methods of additional analyses (e.g., sensitivity or subgroup analyses **targeted pediatric age group(s)**, meta-regression), if done, indicating which were pre-specified.	
Results			
Study selection	17	Give numbers of studies screened, assessed for eligibility, and included in the review, with reasons for exclusions at each stage, ideally with a flow diagram.	
Study characteristics	18	For each study, present characteristics for which data were extracted (e.g., study size, PICOS, follow-up period) and provide the citations.	18a. Provide sample size of pediatric group and sub-groups (if applicable) for each study
Risk of bias within studies	19	Present data on risk of bias of each study and, if available, any outcome level assessment (see item 12).	
Results of individual studies	20	For all outcomes considered (benefits or harms), present, for each study: (a) simple summary data for each intervention group (b) effect estimates and confidence intervals, ideally with a forest plot.	
Synthesis of results	21	Present results of each meta-analysis done, including confidence intervals and measures of consistency.	21a. Report the numbers of included studies with pediatric participants and, where applicable, report the number of events and total pediatric population on which the result synthesis is based. 21b. Provide a description of the direction and size of effect in terms meaningful to those who would put findings into practice in a pediatric population.
Risk of bias across studies	22	Present results of any assessment of risk of bias across studies (see Item 15).	
Additional analysis	23	Give results of additional analyses, if done (e.g., sensitivity or subgroup analyses **for targeted pediatric age group(s),** meta-regression [see Item 16]).	
Discussion			
Summary of evidence	24	Summarize the main findings including the strength of evidence for each main outcome; consider their relevance to key groups (e.g., healthcare providers, users **e.g., children and/or their parents,** and policy makers).	
Limitations	25	Discuss limitations at study and outcome level (e.g., risk of bias), and at review-level (e.g., incomplete retrieval of identified research, reporting bias), **including any limitations arising from pediatric studies that were not available.**	
Conclusions	26	Provide a general interpretation of the results in the context of other evidence **(e.g., evidence from adult studies, preclinical studies and studies based on routinely collected data e.g. Electronic health Record data or Registry data),** and implications for future research.	26a. Provide extent and limits of applicability of the synthesised evidence to pediatric population of interest and describe the evidence and logic underlying those judgements. 26b. Provide implication for research, practice, or policy related to pediatric population where relevant (e.g., types of research needed to address unanswered question in the pediatric population). 26c. Provide a general interpretation of the results in the context of other evidence (e.g., evidence from adult studies, observational studies, and pre-clinical studies). Provide implications for future research, practice, or policy related to the targeted pediatric age group(s).
Funding			
Funding	27	Describe sources of funding for the systematic review and other support (e.g., supply of data); role of funders for the systematic review.	

*modifications text in the original items are bold; extension items are represented in a separate column labelled as PRISMA-Children Potential extension items

### Future directions

First, we recommend that the current PRISMA statement be used to systematically develop a checklist of essential items which researchers should include when designing, conducting and reporting pediatric systematic reviews. Second, it seems time to develop two versions that could be called PRISMA- PC (Protocol for Children) and PRISMA-C (Reporting for Children). The detailed protocol of the planned process for developing new PRISMA-PC and PRISMA-C statements are provided elsewhere [62]. These “extensions” of the current PRISMA statement are aimed to improve the quality of the information reported in pediatric systematic reviews by highlighting the key elements that differ from adult reviews. The “extended” reporting items should be underpinned by evidence for their importance.

The impact of this work will be potentially large. Adherence by systematic reviewers to PRISMA and its “pediatric extensions” will facilitate clarity, completeness, and transparency of pediatric systematic reviews reporting. Explicit descriptions and transparent reporting of these minimum set of items will best serve the interests of all stakeholders and will also reveal any deficiencies in the research if they exist. It is anticipated, given the focus of knowledge synthesis and the requirement by journals for relating studies to existing evidence, that pediatric specific systematic review guidelines would in turn influence the design, conduct, analysis and reporting of primary trials in children. Finally, PRISMA-PC and PRISMA-C guidance will help facilitate the translation of knowledge from the literature to bedside care, thereby improving child health and reduce the burden on the healthcare system.

### Conclusions

Systematic reviews are key tools to enable decision making by healthcare providers and policymakers. The *Preferred Reporting Items for Systematic reviews and Meta-Analysis* - PRISMA-2009 and PRISMA-P 2015 - statements were developed to improve the transparency and quality of reporting of systematic reviews. The lack of guidance in these statements for pediatric-specific methodological features of systematic reviews on child health topics may impair decision-making in child health. In collaboration with members of the PRISMA Group, we will develop a consensus-based checklist of essential items which researchers should include when planning and reporting pediatric systematic reviews. PRISMA-PC (protocol-children) and PRISMA-C (reporting-children) guidance will help facilitate the translation of knowledge from the literature to bedside care and policy, thereby improving child health outcomes and enhancing delivery of care.

## Abbreviations

CONSORT-C: Consolidated Standards of Reporting Trials in Children
MDG: Millennium Development Goals
MNCH: Maternal and Child Health
PRISMA: Preferred Reporting Items for Systematic reviews and Meta-Analysis
PRISMA-C: Preferred Reporting Items for Systematic reviews and Meta-Analysis-Children
PRISMA-P: Preferred Reporting Items for Systematic reviews and Meta-Analysis-Protocol
PRISMA-PC: Preferred Reporting Items for Systematic reviews and Meta-Analysis- Protocol for Children
PROSPERO: Prospective Register of Ongoing Systematic Reviews
RCT: Randomised Controlled Trials
SPIRIT-C: Standard Protocol Items for Randomized Trials in Children
